# A Guide to Investigating Suspected Outbreaks of Mucormycosis in Healthcare

**DOI:** 10.3390/jof5030069

**Published:** 2019-07-24

**Authors:** Kathleen P. Hartnett, Brendan R. Jackson, Kiran M. Perkins, Janet Glowicz, Janna L. Kerins, Stephanie R. Black, Shawn R. Lockhart, Bryan E. Christensen, Karlyn D. Beer

**Affiliations:** 1 Prevention and Response Branch, Division of Healthcare Quality Promotion, Centers for Disease Control and Prevention (CDC), Atlanta, GA 30333, USA; 2 Epidemic Intelligence Service, CDC, Atlanta, GA 30333, USA; 3 Mycotic Diseases Branch, Division of Foodborne, Waterborne, and Environmental Diseases, CDC, Atlanta, GA 30333, USA; 4 Chicago Department of Public Health, Chicago, IL 60604, USA

**Keywords:** mucormycosis, mucormycetes, mold, cluster, outbreak, infections, hospital, healthcare

## Abstract

This report serves as a guide for investigating mucormycosis infections in healthcare. We describe lessons learned from previous outbreaks and offer methods and tools that can aid in these investigations. We also offer suggestions for conducting environmental assessments, implementing infection control measures, and initiating surveillance to ensure that interventions were effective. While not all investigations of mucormycosis infections will identify a single source, all can potentially lead to improvements in infection control.

## 1. Introduction

Mucormycosis is a rare but serious infection that can affect immunocompromised patients in healthcare settings. Investigations of possible mucormycosis outbreaks in healthcare settings have enabled critical insights into the exposures and environmental conditions that can lead to transmission. The purpose of this report is to compile resources and experiences that can serve as a guide for facilities and health departments investigating mucormycosis infections and suspected outbreaks in healthcare settings. We first provide an overview of mucormycosis outbreak investigations and then suggest how the typical steps of an outbreak investigation can be adapted and applied to mucormycosis infections in hospitals.

### 1.1. Mucormycosis Background

Mucormycosis is caused by a group of molds called mucormycetes, which are found in the environment and release spores that are easily aerosolized and dispersed [1]. The most common genera that cause infections in humans are *Rhizopus* and *Mucor* species, but others include *Apophysomyces*, *Rhizomucor*, *Cunninghamella*, *Lichtheimia, Cokeromyces*, and *Saksenaea* [2]. Mucormycosis can affect nearly any part of the body, with rhinocerebral and pulmonary infections caused by inhalation of spores, and cutaneous infections caused by spores entering the skin. Gastrointestinal mucormycosis can occur when contaminated foods or products are introduced to the GI tract. Mucormycetes can infiltrate blood vessels and spread to the brain and other organs through the bloodstream, resulting in disseminated infections. Mucormycosis outbreaks have involved all of these clinical presentations. Risk factors for mucormycosis include immunosuppression, with many infections seen in people who have had stem cell or solid organ transplant, hematologic malignancies, and long-term treatment with corticosteroids [3].

Because mucormycetes grow best in acidic, high-glucose environments, diabetes, especially if poorly-controlled or complicated by ketoacidosis, is also an important risk factor [2]. Some evidence suggests that the number of invasive mucormycosis infections has risen over time, in part because of better recognition and diagnosis, but also because advances in cancer treatment have increased the number of immunocompromised patients most vulnerable to infection [4,5,6,7,8,9,10,11,12,13]. An analysis of US hospital discharge data found that the overall incidence rate of mucormycosis-related hospitalizations doubled from 1.7 to 3.4 per million US residents from 2000 to 2013, representing an average annual increase of over 5%. In 2013, there were an estimated 1080 US hospitalizations (95% CI: 875–1275) with diagnostic codes for mucormycosis [4]. A study of the direct cost of fungal diseases in the United States identified 1140 mucormycosis hospitalizations in 2014 (95% CI: 912–1368), at a cost of $106,655–112,849 per inpatient hospitalization, for a total annual US inpatient cost of $125 million [13].

### 1.2. Challenges of Investigating Mucormycosis

Exposure to mucormycetes can cause outbreaks among at-risk patients in healthcare settings, with infections linked to construction, water leaks, insufficient air filtration, non-sterile medical supplies, deficient device reprocessing, dental procedures, contaminated linen, contaminated food and supplements, and other sources of mold. These infections are often devastating, with reported mortality rates around 50% in some studies [2,14,15]. The sources of mucormycosis infections in healthcare are particularly challenging to investigate, in part because patients may have complicated medical histories with many different exposures, and the incubation period for non-cutaneous mucormycosis is not well established. In addition, the diagnostic criteria needed to distinguish invasive infection from colonization are complex, and mycology laboratory capacity for species determination is variable. Molecular methods can be very useful for species determination, but have only recently been applied to support epidemiologic linkages between patient and environmental mucormycete isolates in order to identify outbreak sources [16,17,18].

Because mucormycosis is neither nationally notifiable nor reportable in any US state, there is no public health surveillance system to monitor infections, so it is difficult to discern whether a hospital’s infection rate is above average. Mucormycosis is a rare infection even among high-risk patients, with a 12-month incidence of <1% in one cohort of hematopoietic cell transplant (HCT) and solid organ transplant recipients during 2001–2006 [19] and an incidence of 2% in a cohort of HCT recipients followed for up to 30 months after transplant during 2006–2011 [20]. Because infections are infrequent even in large transplant centers, it can be difficult to determine whether an apparent increase at a hospital is because of chance, a changing patient population, or a new source of mold. 

A final challenge in investigating mucormycosis outbreaks is that mold spores can be found in many different environmental sources, including air, dust, soil, and on surfaces, and may not be visible. Infection control assessments often identify multiple areas for improvement, and it is rare to identify a single point source of mold that is epidemiologically linked to cases. Because many outbreaks are multifactorial and require hospitals to take several mitigating actions simultaneously, it can be impossible to assess the effects of individual interventions. 

## 2. Components of an Outbreak Investigation

### 2.1. Verify the Diagnosis and Define a Case

The most widely used and accepted case definition for invasive fungal infections was developed for use in clinical trials among transplant patients by the European Organization for Research and Treatment of Cancer (EORTC) and the National Institute of Allergy and Infectious Diseases Mycoses Study Group (MSG) [21]. CDC has added attributes of mucormycetes to make this definition specific to mucormycosis (Table 1). While the definition of proven invasive mucormycosis is straightforward, few infections have sufficient evidence to meet this definition. Many other infections are considered probable cases. However, the probable definition is highly complex, difficult to apply objectively, and specific to pulmonary, sinonasal, and central nervous system (CNS) presentations, meaning it does not address cutaneous and wound infections. For these reasons, mucormycosis case definitions during outbreaks have varied based on the presentation and availability of data, but all require histopathology or culture along with host and clinical criteria. Although cultures can be less invasive and easier to obtain, histopathologic evaluation of tissue specimens can be key to confirming a diagnosis. Histopathology results can be challenging to include in a mucormycosis case definition, because it can be difficult to search the free text of pathology reports. However, omitting histopathology results during case-finding can result in inadvertently missing cases. For example, in a review of 169 EORTC/MSG proven and probable cases, 160 had histopathologic findings positive for typical mucormycete hyphae. Of the 169 cases, 52 (31%) did not have an accompanying positive culture so would not have been identified without histopathology [3].

#### 2.1.1. Cutaneous Mucormycosis Case Definition

Case definitions for cutaneous infections have included soft tissue infections with evidence of mucormycosis, such as positive fungal culture and observation of broad pauciseptate hyphae with branching at right angles on microscopy, mainly at the periphery of the skin lesion [22]. Case definitions for cutaneous infections may also include common presentations, such as rapid progression from erythema to necrosis. In an investigation of cutaneous mucormycosis infections after a tornado, a case was defined as (1) a necrotizing soft-tissue infection requiring antifungal treatment or surgical debridement and (2) positive fungal culture or histopathology and genetic sequencing consistent with a mucormycete [23]. 

#### 2.1.2. Laboratory Confirmation

Laboratory confirmation of mucormycetes in patient specimens is a central part of all case definitions above, although mycology laboratory capacity varies by hospital. Those with mycology expertise should identify species of suspected mucormycete cultures, although species identification of mucormycetes can be difficult, and misidentifications occur commonly in clinical laboratories. Where capacity is limited, cultures may be sent out to reference diagnostic laboratories. In some states, public health laboratories can assist with diagnostics. The CDC offers a comprehensive fungal diagnostic service and can typically receive suspected mucormycete isolates when state public health partners are involved [24]. Polymerase chain reaction (PCR) testing can identify mucormycetes in some formalin-fixed paraffin-embedded biopsy specimens with histopathologic evidence of fungal hyphae at CDC [25,26]. Some other institutions also perform PCR on tissue and tissue blocks for a fee. However, this technique is heavily dependent on source, abundance, and quality of the specimen. Some analyses have suggested that PCR testing to detect mucormycete DNA in serum can be useful in screening at-risk patients, with faster detection and results that are concordant with culture or mucormycete species identified by PCR from tissue [27,28]. However, commercially available serum PCR test kits for mucormycetes are not currently available, so without prior validation, these tests would not conform with US Clinical Laboratory Improvement Amendments (CLIA) or College of American Pathologists (CAP) standards. Identifying the presence of a mucormycete by immunohistochemistry can be useful during the initial investigation, even in the absence of a definitive genus or species [29]. 

### 2.2. Evaluate Hospital Trends in Mucormycosis and Other Mold Infections

Because mucormycosis is rare even among patients at the highest risk, investigation is warranted even when it is unclear whether an increase in the number of infections represents a true outbreak, particularly if cases appear to cluster in time or place or among patients who share other exposures. For example, an increase from two to four cases per year at a single hospital could be because of chance or could signal that a new source of mucormycetes is present in the facility, leading to infections. 

#### 2.2.1. Assess Trends for All Mold Infections

To assess changes in infection rates over time, hospitals can begin by examining trends in all invasive mold infections (for list of pathogenic molds, see Supplementary Materials S1), including *Aspergillus*, which causes infections more frequently than mucormycetes. Tracking *Aspergillus* infections may help to identify problematic exposures, because like mucormycosis, these molds are transmitted via airborne spores. Facilities may consider looking for infections in the previous 12 to 24 months by reviewing microbiology records for positive fungal culture results, and, if possible, reviewing histopathology specimens with evidence of tissue invasion by fungal hyphae. A spreadsheet of specimens (sometimes called a line list) should be created that includes basic information about each culture or pathology specimen, including a patient identifier, date collected, and organism identified. This initial line list can be expanded to include additional clinical details as the investigation progresses (downloadable sample line list, Supplementary Materials S2). 

**Figure 1:**
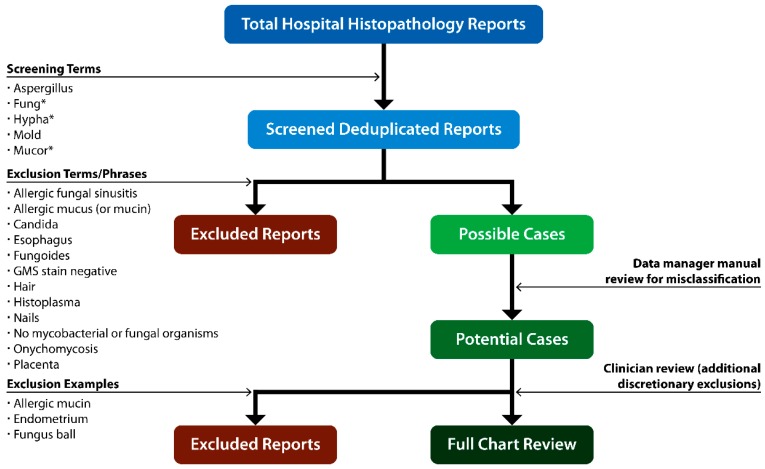
Invasive mold surveillance histopathology case-finding algorithm to search the free text of pathology reports.

One strategy to identify infections from pathologic records is to use an algorithm to search the free text of histopathology reports (Figure 1). Real-time case finding can be conducted in collaboration with pathologists, who can remain watchful for evidence of tissue invasion and angioinvasion and notify investigators as it is identified. One limitation of this approach is that pathology reports rarely include mucormycete species; often they report only that hyphae or fungal elements were observed. Key terms, such as broad, ribbon-like, aseptate or pauciseptate, can be useful.

#### 2.2.2. Using Denominator Data to Calculate Incidence

Because the incidence of mucormycosis infections appears to have increased with the growing population of vulnerable patients, calculating incidence using the number of vulnerable patients as the denominator may be more informative than relying solely on the number of infections to assess hospital trends. For example, incidence can be calculated using total number of infections per 100 transplants per year, or per 1000 patient-days on a transplant unit [7]. Some hospitals investigating possible outbreaks of mucormycosis have worked with other transplant centers to track infection rates over time using a standardized case definition. This can help to establish whether rates of infection are unusually high at any particular hospital, or might vary by season or weather patterns in the region. Outside of the outbreak setting, prospective mucormycosis surveillance could provide valuable information about typical rates among different patient populations and about geographical and seasonal differences.

#### 2.2.3. Seasonality

Several studies have investigated whether mucormycosis infections may have seasonal patterns. Three studies from the eastern Mediterranean, two in Israel [30,31] and one in Lebanon [32], found that most mucormycosis infections presented during August through December, peaking at the end of the hot, dry summer season. In Seattle, a study found 5.7 invasive mold infections per 10,000 patient-days occurred in summer, compared with 1.3 per 10,000 in the spring during 2012–2015 [33]. While season should be considered in examining clusters of infections, small numbers over several years at any given hospital make it difficult to interpret the importance of seasonality. These studies may reflect publication bias if investigators who notice clustering by time are more likely to publish or mention seasonality as a risk factor than those who do not.

### 2.3. Notify and Involve Key Partners

Mucormycosis investigations require a multidisciplinary team. Optimally, the team should include those with clinical expertise, as well as those with expertise in case-finding, environmental assessment, and infection control. Effective response to a suspected outbreak thus requires early communication and close collaboration among infection prevention, facility maintenance, construction, environmental services, nursing, infectious disease, pathology, hospital administration, and media relations. When beginning an investigation of a suspected outbreak of mucormycosis, we recommend convening a meeting with representatives from each of these groups. In the sections that follow, we discuss specific actions and resources that may be useful for each of these representatives. US states require reporting of outbreaks in healthcare settings to state or local health departments, which are often able to offer consultation, laboratory support, and on-site assistance with investigations of mucormycosis.

In addition, hospitals should notify patients who develop mucormycosis as a suspected result of an infection control breach, and should also notify patients at elevated risk during mucormycosis outbreaks that are likely to be healthcare-associated. Dudzinski et al. [34] recommend informing patients of known infection control breaches that increase the risk of injury or harm, even if the chance of patient harm is extremely low. The authors argue that these disclosures should be the norm unless a strong, ethically justifiable case can be made not to disclose. Although there may be legal risks to the institution, disclosing potential outbreaks may increase public perception of the hospital’s integrity and reduce the chance that the public will view the hospital as having withheld information from patients. One recent study at four Massachusetts hospitals found that malpractice claims did not increase following implementation of a communication-and-resolution program through which the hospitals communicated with patients about adverse events and took responsibility when appropriate [35]. Hospitals should consult their legal counsel, communications experts, and state public health department for additional assistance. 

Timely notification of hospital staff and patients can:

- Encourage patients to seek care early if they have concerning symptoms, potentially leading to more timely diagnosis;
- Improve case-finding;
- Provide opportunity to educate patients on ways to avoid exposure to mold, both in the hospital and community;
- Remind patients, visitors, and clinicians about important of infection control interventions;
- Build patient and public trust through transparency.

For additional guidance, CDC has a patient notification toolkit for healthcare facilities, which includes resources such as sample patient letters [36]. 

### 2.4. Determine Exposures of Interest and Relevant Time Period

Once an investigation has begun and cases identified, the first step toward understanding the patients involved and identifying potential common exposures is a thorough description of the infections. This description includes the classic epidemiologic triad of person, place, and time. We describe tools for this analysis below.

#### 2.4.1. Epidemic Curve 

**Figure 2:**
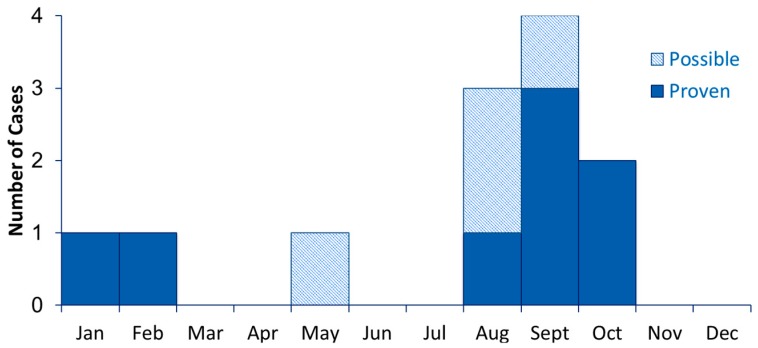
Sample epidemic curve, showing the number of proven and possible cases by month.

Because mucormycosis infections are rare, an epidemic curve (Figure 2) may be most useful when including cases from previous years. It may also be helpful to distinguish proven cases from those that are probable and possible. Because the initial signs and symptoms of mucormycosis are nonspecific and common in vulnerable patient populations, it may be difficult to assign the date of infection onset. When medical records are the only available source of information, the date on which the first diagnostic specimen was obtained, rather than symptom onset, may be more reliable [37]. 

#### 2.4.2. Line List and Case Reviews

Key details that should be collected on a line list for each patient include:

- Age;
- Admission date(s), as well as discharge dates for previous hospitalizations within several months of suspected onset;
- Prior admissions and outpatient visits;
- Patient risk factors and underlying conditions (e.g., diabetes mellitus, hematologic disease, hematopoietic stem cell transplant, and prolonged neutropenia);
- Immunosuppressive medications (e.g., corticosteroids, cancer chemotherapy, biologics, and other immunotherapy);
- Signs and symptoms of fungal infection, with approximate dates of onset;
- Histopathology specimen collection date(s) and findings;
- Culture date(s), specimen source, and species;
- Antifungal medications given with dates and indication (prophylaxis versus treatment);
- Unit and room assignments with dates;
- Invasive disease versus colonization by EORTC/MSG definition or clinician determination;
- Devices, procedures, lines, and surgeries;
- Outcomes, including date of discharge or death.

#### 2.4.3. Hospital Map

Visualizing the location of patients in relation to events such as construction, renovations, maintenance work, and water and air leaks may help to identify shared exposure. Maps of the air handling systems with relative pressures between units, hallways, and rooms can be overlaid with patient information to determine whether locations shared by cases were supplied by the same air handlers. 

#### 2.4.4. Timeline of Patient Locations

**Figure 3:**
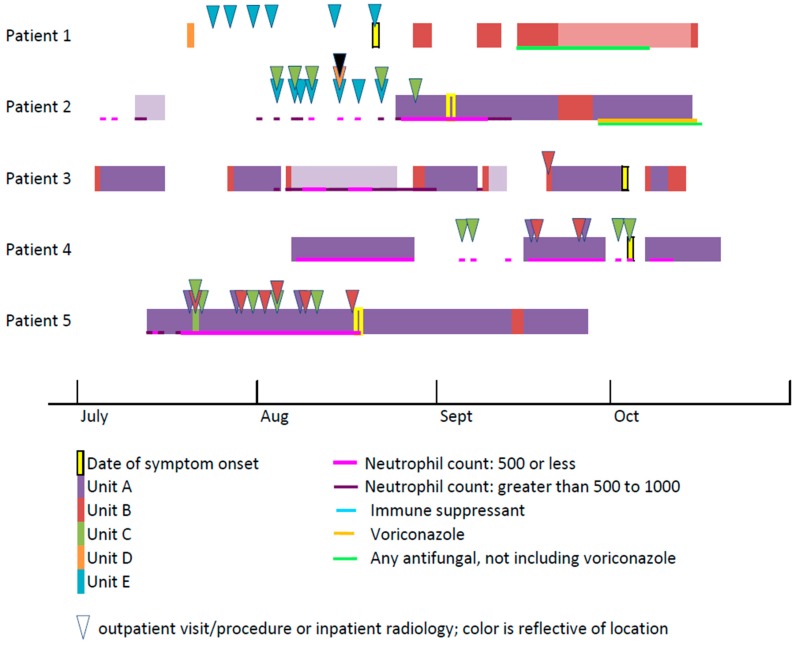
Sample timeline of inpatient locations and outpatient visits. Each of the five lines represents a different patient. Outpatient visits are depicted with a triangle color-coded by location. Days in each inpatient unit are depicted by color-coded bars. The lines under the bars show days the patient was immunosuppressed and received antifungal medication.

The frequent movement of patients within a healthcare facility can complicate mucormycosis investigations. In investigations with complex patient histories, it can be helpful to create a timeline of each patient’s location, including room and unit, in the months prior to diagnosis (Figure 3). This may help to clarify places and times of potential shared exposure in the hospital. 

Considering whether transmission more likely occurred in the healthcare setting or in the community is important. Because the incubation period for mucormycosis is not well established, no standard definition of healthcare-associated mucormycosis exists. However, some evidence is available about the incubation period of cutaneous infections. A review of cutaneous mucormycosis infections found that the median incubation period for 168 patients with a single percutaneous exposure was 8 days (interquartile range: 5–16 days) [38], similar to a study of mucormycosis infections following a tornado, where time from injury to first positive culture ranged from 6–24 days [39]. These findings suggest a relatively short incubation period for cutaneous mucormycosis, although the incubation period may differ by factors including the patient’s immune status and other risk factors. 

The incubation period for rhinocerebral and pulmonary mucormycosis is not well established, making it difficult to establish a time period in which to identify relevant exposures. Patients at high risk for healthcare-associated mucormycosis may have had numerous procedures and inpatient stays in many rooms on different units. One strategy that may be used when evaluating patients with long hospitalizations and multiple healthcare exposures is to begin by focusing on exposures in the 14 days before suspected infection onset, then expanding to a longer window before infection if common exposures to possible sources of mucormycetes are not evident. Consider using a longer window for infections when the time of onset is difficult to pinpoint. For example, in transplant patients, the time since transplant can guide exposure identification.

### 2.5. Develop Hypotheses

The descriptive epidemiology described above can provide clues to common exposures among patients and enable hypothesis generation. In Table 2 and the section below, we review a set of outbreak reports that highlight these associations. Mucormycosis outbreaks have been epidemiologically associated with a diverse set of exposures. The initial presentations of infections may offer insight into the locations where spores were introduced. For example, peritonitis infections were linked to peritoneal dialysis and a bladder infection to a bladder catheter. In cutaneous infections, the first lesions often appeared where the skin made contact with contaminated products including bandages, adhesives, and fabric. Gastrointestinal infections were associated with contaminated oral supplements and medications. Infections associated with a contaminated organ often began at the site of transplantation, while others that began near the incision site were suspected to be surgical site infections caused by spores in the hospital environment. Pulmonary infections were associated with exposure to airborne spores, such as during hospital renovation. However, a single source may cause infections with multiple presentations. For example, contaminated linens, as well as use of a negative pressure room for immunocompromised patients, were associated with both pulmonary and cutaneous presentations. 

However, outbreak investigations linking infections to a single exposure are the exception rather than the rule; most investigations lack sufficient evidence to attribute a mucormycosis cluster to one point source. CDC records include 17 mucormycosis-related consultations from January 2005–April 2019, of which six resulted in an epidemiologically robust attributable exposure [17,39,40,41,42,43]. Publication bias toward outbreaks with identification of a single source might contribute to an under-appreciation of the difficulty in determining the source of mucormycetes. Implicated exposures in published reports are likely not representative of the true distribution of outbreak sources. 

#### 2.5.1. Non-Sterile Products

##### Bandages, Patches, Tape, and Adhesives:

Cutaneous mucormycosis infections have been associated with the use of non-sterile bandages, patches, tape, and adhesives, with the first necrotic lesions usually appearing underneath the product. Between 1977 and 1978, elastic adhesive bandages were implicated in an outbreak of more than 20 *Rhizopus* infections in at least 11 hospitals [44,45,46,47,48]. The time from adhesive application to development of lesion was seven to nine days for most patients [46,47,48,49,50]. The bandage company did not guarantee sterility of the product at the time of the outbreak, and *Rhizopus* species were isolated from unused or partially used bandages in multiple hospitals [44]. 

Two hospitals, one in Belgium and one in Pakistan, had outbreaks of *Lichtheimia corymbifera* from non-sterile bandages. Both hospitals subsequently recommended using only sterile bandages for deep, traumatic, and extensive wounds, including burns [51,52]. Patients in burn units may be at particularly high risk from non-sterile products, due to their extensive wounds. In the Belgian hospital, 7 of 27 patients admitted to the burn unit in a 6-month period were found to be colonized with *L. corymbifera*. Of those seven patients, five developed infections and three died. A third hospital in France used real-time PCR to test non-sterile bandages used in the burn unit, detecting *Lichtheimia* spp. in six out of eight crepe bandages and four out of six elasticized bandages [92]. The hospital documented mucormycosis in 17 burn patients between 2005 and 2016, of which 14 were *Lichtheimia* spp. 

Ulcers caused by mucormycosis found under tape used to secure an endotracheal tube were reported at two different hospitals, although neither tape was tested for contamination [53,54]. As a result, one of the hospitals recommended securing the endotracheal tube with surgical tubing or fabric fastener straps and routine skin checks for earlier diagnosis.

Adhesives used to secure ostomy bags can also be considered as a source of infection, particularly if they are infrequently changed. In 2005, two patients at the same hospital developed skin and soft tissue infections with *Rhizopus arrhizus* (*oryzae*) at the site of recent surgery to create a stoma [40]. The adhesive used to secure the ostomy bag to the abdomen contained karaya gum, a non-sterile product produced from tree sap that is commonly used as a skin barrier in ostomy supplies. *Rhizopus arrhizus* was isolated from 10 of 18 unopened karaya ostomy bags at the hospital, and from 3 of 29 karaya ostomy bags from other hospitals. The median time to first ostomy bag change was 8.5 days for the case-patients, compared with 1.5 days for patients who underwent the same procedure but did not develop an infection; the hospital replaced karaya gum with a synthetic product and recommended more frequent changes. An earlier case report describes an infection that developed around an ileostomy, but the ostomy supplies were not cultured [93].

Other cutaneous mucormycosis infections have been linked to adhesive patches used to attach a temperature probe [55] and heart monitor [56] in preterm infants, nitroglycerin patches applied at home [57], and non-sterile cotton fabric used under a cast [58]. 

##### Linen:

The investigations described below suggest that investigators of possible mucormycosis outbreaks should consider a comprehensive evaluation of hospital linen washing, transportation, storage and use, from the laundry facility to the patient. Fungal outbreaks from linen have been attributed to contamination of laundered textiles at off-site laundries, during transportation, and during storage in hospitals [94]. An investigation of five fatal cases of cutaneous mucormycosis, three with *Rhizopus delemar*, at a children’s hospital over 11 months found that clean linens and clean linen delivery bins from the off-site laundry were contaminated with the same species [17]. The children were in different units of the hospital served by different air handlers and shared few common products or procedures, leading investigators to focus on linen as a potential source. 

In another investigation of skin and soft tissue infections that began in the axillae of four immunocompromised patients on the same unit, the facility recovered *Rhizopus* spp. from multiple laundry carts. The outbreak stopped when the hospital changed its practice from cleaning carts only when they were soiled to a routine cleaning schedule, as recommended by the Healthcare Laundry Accreditation Council [59].

Another hospital found widespread contamination of hospital linen after six patients in less than two months had infections with *Rhizopus microsporus* [18]. Two infections were cutaneous, three were pulmonary, and one was both cutaneous and pulmonary. The investigators cultured mucormycetes from 12% of laundered linens at the hospital, with no mucormycetes cultured from linens at control hospitals. An inspection of the hospital’s laundry contractor found poor ventilation, warm and humid indoor air, and thick layers of dust on surfaces, suggesting that the linens may have become contaminated after washing. Investigators suggested that the patients’ close proximity to bed linen and clothing could cause both cutaneous infections by direct contact with contaminated linen and pulmonary infection through inhalation of spores. 

In response to a mucormycosis outbreak where linens were one suspected source, investigators conducted an audit of 15 transplant and cancer hospitals and found seven (47%) centers had freshly laundered healthcare linens contaminated with mucormycetes. Three (20%) of the 15 centers failed to meet hygienically clean standards for mucormycetes [95]. In total, 14% of healthcare linens were found to have mucormycetes. Visibly soiled linens or carts, higher maximum temperatures, and relative humidity near laundry were associated with contamination found by sampling. The investigators did not have clinical data on fungal infections at the participating hospitals, so were unable to assess whether contamination found by sampling was associated with higher risks of patient infections.

##### Wooden Tongue Depressors: 

Because wood is a natural substrate for mucormycetes, any wooden products used in patient care should be investigated. Wooden tongue depressors were associated with infections in five adults diagnosed with gastric mucormycosis. *Rhizopus microsporus* was detected on wooden tongue depressors used to prepare oral medications given through a nasogastric catheter [60]. Given several previous reports of laboratory contamination caused by non-sterile wooden tongue depressors, spatulas, and sticks used to collect and prepare stool specimens [96,97,98], the hospital recommended against using wooden tongue depressors in the ICU and hospital, especially in critically ill immunocompromised patients. Four cutaneous infections among infants in a neonatal intensive care unit were also linked to wooden tongue depressors used as splints for intravenous and arterial cannulation sites [61].

##### Medications, Supplements, and Food:

Infections have also been linked to contaminated medications, supplements, and food. In October 2014, a premature infant died of gastrointestinal mucormycosis caused by *Rhizopus* *oryzae* following the use of a probiotic powder intended to contain three bacteria, *Bifidobacterium lactis*, *Streptococcus thermophilus*, and *Lactobacillus rhamnosus*. Testing showed the powder was contaminated with *R. oryzae* [43]. An earlier case report describes a hepatic infection in a bone marrow transplant patient who had taken an oral *Lactobacillus* supplement [62]. The PCR analysis showed an identical banding pattern between the *Mucor indicus* isolates from the patient’s liver aspirate and the supplement. 

In response to an outbreak of seven intestinal infections over six months, a team of investigators at a hospital in Hong Kong found that the infections were associated with contaminated allopurinol tablets and pre-packaged food [63]. While sequencing showed that the patient isolates clustered closely with isolates from both the allopurinol and food, investigators thought the allopurinol tablets were more likely the cause, due to the higher mean viable fungal counts. 

#### 2.5.2. Procedures, Devices, and Organs

##### Catheters and Tubes:

Mucormycetes can be transmitted during insertion of catheters and lines. Mucormycosis infections were reported in an adult patient with a permanent bladder catheter [64] and in two infants, one where a necrotic lesion formed near a periumbilical cutaneous catheter site and another where spores appeared to enter through a chest tube site [65]. In another infection, investigators hypothesized that the spores were introduced to a preterm infant’s gastrointestinal tract through an orogastric tube [66]. Dialysis catheters are another potential source of infection [67,68,69,70]. In one case report of peritonitis in a patient receiving peritoneal dialysis, the patient’s peritoneal fluid and catheter cuff cultured positive for the same mucor species [67]. In another, the dialysis catheter sterilization procedure may not have been effective against spores [68]. 

##### Dental Procedures:

Another possible exposure to consider, particularly for mucormycosis of the maxilla or sinuses, is a previous dental procedure, such as an extraction, that could allow spores to enter where the tooth was removed. In India, cases of rhino-orbital mucormycosis were reported in two patients, one diabetic, who had dental procedures 8 and 12 days before hospitalization [71]. Case reports from Hungary [72], California [73], and Bahrain [74] also describe rhinocerebral mucormycosis in diabetic men, who were admitted three to five days after dental procedures. Other reports describe mucormycosis infections of the maxilla [75] and mandible [76] following tooth extraction.

##### Cardiac Surgery: 

Mucormycosis infections that appear to begin near a surgical site may be the result of contamination in the operating, recovery, or patient rooms. Infections have been found six days following explantation of left ventricular assist devices (LVADs) [77] and up to six weeks after implantation of prosthetic valves [78,79,80]. One patient developed infection seven days after cardiac catheterization and bypass surgery around the sternal wound and chest tube site, with the mode of transmission suspected to be airborne contamination of the surgical site [81]. The hospital investigating infection following explantation of the LVAD found air samples in the operating room and intensive care unit negative for spores, but could not rule out undetected or transient contamination [77].

##### Insulin Pumps and Finger Sticks:

Because patients with poorly controlled diabetes mellitus are at higher risk for mucormycosis, any exposure that breaks the skin should be assessed as a potential source of spores. Infections have been documented in diabetic patients that began at the site of insulin infusion pump insertion [82], daily insulin needle punctures [83,84], and finger sticks for blood glucose self-monitoring [85]. These cases underscore the importance of injection safety practices among diabetic patients, including cleaning and disinfecting the skin before injection and not using equipment contaminated by soil or reuse.

##### Medical Devices:

Medical devices that have not been sufficiently sterilized may be contaminated with spores. One hospital ended an outbreak of postoperative bone mucormycosis among three patients after improved sterilization of screws used in ligament reconstruction [86].

##### Organ Transplant:

Mucormycosis among organ transplant recipients should be investigated when multiple case-patients share a common donor, when the donor or organ might have been exposed to mold, or the donor had evidence of a fungal infection. Mucormycosis infections have been reported following transplants with solid organs that may have become contaminated with mold at the time of the donor’s death, or during harvesting, shipping, or transplantation [99]. For example, renal infections were reported in two patients who both received a kidney from a near-drowning victim after a motor vehicle crash [41]. Several other investigations of mucormycosis infections following transplants concluded spores were not donor-derived and were more likely introduced during the transplant surgery [100,101,102,103].

#### 2.5.3. Environmental Sources: Air, Dust, Water, Soil

##### Air Filtration and Intakes:

Insufficient air filtration and air intakes that are near the ground, outdoor construction, or exhaust, may also be associated with mucormycosis infections. A hospital investigating four cases of mycotic endocarditis among open-heart surgery patients in a single year, including one with *Mucor* sp., isolated *Mucor* sp. and *Aspergillus* sp. in dust from an air conditioner duct and in air samples. The team found that the air filter used was likely insufficient to trap spores [87]. 

At a second hospital, a cluster of three sinus infections in leukemia patients hospitalized on the same hematology unit was also suspected to be caused by poor air filtration [88]. The hospital unit’s air intake was between 10 and 20 cm from the ground near a trash compactor in an outdoor yard, below the 6 feet (1.8 m) above grade recommended by the American Society of Heating, Refrigerating and Air-Conditioning Engineers [104]. Air and surface samples inside the unit were heavily contaminated with *Rhizomucor pusillus*, as were the intake unit and the yard. Another air conditioning installation at the same hospital had fewer mucormycetes and no *Rhizomucor pusillus*; no mucormycetes were detected in air samples from two comparison hospitals in the same city. 

##### Negative Pressure Rooms:

Negative pressure rooms, which are used to isolate patients with an airborne infection, have been identified as risk factor for mold infections when used for immunocompromised patients. In contrast to positive pressure rooms that protect immunocompromised patients by minimizing infiltration of unfiltered air, negative pressure rooms are intended to prevent the spread of tuberculosis or other airborne diseases and produce a lower pressure in the room compared with the corridor or anteroom. Depending on the airflow from the corridor or anteroom, negative pressure in these rooms may pull in air, as well as fungal spores, through small gaps or crevices in the walls or ceiling. Investigators concluded that a cluster of three probable and one suspect mucormycosis infections were likely caused by the use of a negative pressure room for immunocompromised patients [42]. All patients received care in the only negative pressure isolation room on a 20 bed cardiothoracic unit, although none had a medical condition requiring negative pressure isolation. The negative pressure room was adjacent to a carpeted hallway and a room for visitors. Frequent staff and visitor use of the door could have allowed spores to enter the patient room. 

##### Construction:

Hospital construction, inside or outside the facility, may result in dispersal of dust, aerosolization of spores, and contamination of air handlers, leading to an increased incidence of fungal infections [105,106,107]. During 1976–2014, 28 published reports linked hospital construction to fungal disease outbreaks, mostly of Aspergillosis [108]. Many found contamination of airflow systems and inadequate barriers at construction sites. One hospital reported mucormycosis and *Aspergillus* infections in premature infants on a neonatal intensive care unit (NICU) adjacent to hospital construction, with inadequate barriers that allowed a higher density of mold spores measured in the unit than a comparison area without construction [89]. Investigators identified the major source of mold as dust above a false ceiling. 

##### Water Leaks and Water Damage: 

Water leaks have been identified as a potential source of mold exposure. In one hospital outbreak, two children who developed mucormycosis infections on a pediatric oncology unit were in beds closest to a linen closet that shared a wall with a leaking shower [90]. One developed brain lesions, and the other had an infection at the site of an intravenous cannula. Testing by both air sampling and settle plates showed a high concentration of mucormycete and *Aspergillus* spores near the water-damaged wall, while the spore concentration was zero at comparison sites near the ends of the unit. The spore counts dropped to zero a week after remediation of the leak and wall with mold.

##### Plants:

Because mucormycetes are found in soil, any patient exposure to plants, especially in a healthcare setting, should be considered among possible sources of infection during an investigation. One case of cutaneous infection with *Apophysomyces elegans* was identified in a non-insulin dependent diabetic man after an allergy test that included application of a petal, stem, and leaf from a snapdragon plant to his unbroken skin under a sterile dressing [91]. The man reported pain five days after application, and on the seventh day developed blisters at the site of application. Samples from the snapdragon flowerbed contained multiple fungal species including *Cunninghamella* species but not *A. elegans*; samples of the adhesive did not grow mold or fungi. Investigators hypothesized that the flower was likely contaminated by mold from the soil and recommended washing plants and using less occlusive dressings for future allergy tests, although the efficacy of these interventions is unknown. 

#### 2.5.4. Other Exposures

Case reports have described infections acquired in the community by injection drug use [109], motor vehicle accidents, burns, stings and bites, floods [110], tsunamis, tornadoes [111], and contaminated food. In 2013, >200 people reported gastrointestinal symptoms following ingestion of a commercial yogurt brand contaminated with *Mucor circinelloides* [112], with one immunocompromised patient developing a rhinocerebral infection days after eating the contaminated brand [113].

### 2.6. Conduct Environmental Assessments

Environmental assessments are critical in developing hypotheses in response to a suspected mucormycosis outbreak. These assessments include an examination of the facility with experts in infection prevention and facility staff who oversee building, heating, ventilation, and air conditioning systems (HVAC), construction and maintenance, environmental cleaning, and linen transport and storage. Facility records can also help to identify lapses in scheduled maintenance, construction, and repair history, and other events that may have created environments that support mold growth. Investigators can check any reports to the facilities or maintenance department about possible mold, including any mold referred for remediation. Visual and records-based environmental assessments should typically be performed before conducting sampling. To guide systematic environmental assessments for investigations of mold infections in healthcare settings, the CDC developed a checklist [114]. 

The checklist (Supplemental Materials S3) covers the following topics:

- General inspection for mold, leaks, dirt
- HVAC systems
- Environmental cleaning
- Construction and maintenance
- Linen

A second checklist to assess healthcare linen in more detail is published by the Healthcare Laundry Accreditation Council, a US organization that inspects and accredits laundries serving healthcare facilities [115].

In addition to visual inspection, review of facility records can also help investigators identify possible sources of mold. Records to review include:

- Facility work orders for routine maintenance and repairs
- Infection Control Risk Assessments (ICRAs) conducted before starting construction
- Indoor air temperature and humidity logs, including any days when humidity exceeded 60%
- Dates of air filter changes
- HVAC system, filters, and fans maintained according to manufacturer instructions
- Frequency and method of filter performance
- Air flow monitoring records

#### Protective Environments

The CDC’s Guideline for Isolation Precautions details protective environments needed for allogeneic hematopoietic stem cell transplant (HSCT) patients [116]. The room should have positive pressure of ≥2.5 Pa relative to the corridor, central or point-of-use HEPA filters capable of removing 99.97% of particles ≥0.3 μm in diameter for supply (incoming) air, and at least 12 air changes per hour. Protective environments also should not contain dried or fresh flowers, potted plants, carpets in hallways or patient rooms, or upholstered furniture. The guidelines recommend that hospitals minimize the length of time that patients requiring a protective environment are outside their rooms. During construction, hospitals should provide respiratory protection, such as an N95 respirator, for patients who can tolerate a respirator when they need to leave the protective environment.

### 2.7. Evaluate Hypotheses Epidemiologically

Once hypotheses have been developed from the epidemiologic and environmental review, investigators can evaluate the proportion of infected patients with each exposure before onset. If possible, it may also be useful to conduct an analysis limited to proven cases. 

Although some studies have used cohort and matched case-control designs to evaluate whether patient risk factors such as neutropenia are associated with invasive fungal infection incidence or mortality [5,117,118,119,120,121,122,123], few have used cohort or case-control studies to evaluate whether particular environmental exposures are associated with infections. The absence of such studies from the literature is likely because the number of infections in any cluster is often small, and in many published investigations, all case-patients shared a clear common environmental exposure, making formal hypothesis testing difficult or unnecessary. 

Nonetheless, epidemiologic analyses, while not necessary in all investigations, can add to the weight of evidence about exposures that increase patient risk and inform recommendations for patient care. A study of infections at the site of ostomy bags compared median times between ostomy bag changes in a cohort of patients following stoma creation and found a longer time before bag change in patients who developed infection than those who did not [40]. A cohort study assessing risk of postoperative bone mucormycosis among patients with ligament reconstructive surgery found lower rates among patients undergoing surgery conducted after implementing a device sterilization policy than among patients who underwent the procedure before the change [86], and a team in Hong Kong used statistical analysis to identify the intake of allopurinol tablets and pre-packaged foods as risk factors for intestinal infection [63]. 

### 2.8. Consider Whether Environmental Sampling Could Aid the Investigation

Healthcare facilities are often inclined to begin environmental sampling early in an investigation. While environmental sampling may aid an investigation, it can also be expensive and misleading. Any environmental sampling should be part of a larger building assessment that includes an inspection for moisture incursion and potential sites of mold growth [124,125]. An environmental sample positive for mold can identify potential targets for remediation but cannot be interpreted to indicate a definitive transmission route in the absence of an epidemiologic link to patients. Conversely, a negative result indicates only that spores were not present at the time of sampling using a given method and cannot be used to rule out the presence of mucormycetes. Environmental conditions might change between exposure and sampling, which could result in negative findings.

Molecular epidemiologic tools such as comparing DNA in isolates from patients to isolates from the environment can be helpful in evaluating epidemiologic hypotheses. Some investigators have used whole genome sequencing (WGS) to assess relatedness of isolates [126], but there are limitations of this approach. Because the genomes are complex and there are few good scaffolds for assembling mucormycete genomes, sequencing may not be completed quickly. Another potential problem is that sequencing requires control isolates for comparison that are geographically and temporally related to isolates from cases, which can be difficult to obtain. In addition, many outbreaks of mucormycosis involve multiple species, a scenario when WGS is not useful. Finally, the results of WGS need to be interpreted with caution. Because environmental samples can contain different species and strains of mucormycetes, identifying similar genetic sequences in patient and environmental samples does not prove they are linked. Identification of environmental isolates with dissimilar sequences to clinical isolates cannot rule out a particular source or exposure.

Thus, we recommend considering environmental sampling only after examining shared exposures between cases and conducting environmental assessments including visual inspections and record review. Information obtained during case interviews can focus the environmental evaluation on potential areas of exposure. Environmental sampling methods can include the use of settle plates and spore trap samples followed by microscopic identification. These methods can often be problematic for mucormycetes as a single spore can overgrow an entire plate in less than 24 h, crowding out other species and making the extent of contamination difficult to determine. Combinations of sampling methods can be most powerful, as each method has weaknesses. For example, spore trap samples often cannot detect large, non-airborne spores, or small spores that are not pulled into the sampler due to the short sampling times. Hard surfaces can be sampled using swabs, and dust can be collected from soft surfaces like linen and carpet using small vacuums.

Comparing spore counts and species profile between indoor and outdoor samples can be useful in interpreting results. The concentration of mold in outdoor samples should exceed indoor samples, and the species profiles should be similar. The presence of a predominant species in indoor air that is not found in outdoor air may indicate an indoor source that needs remediation. 

Because little is known about spore counts on linen or in air in the absence of an outbreak, environmental sampling to test results for the hypothesized exposure against a comparison group may be most informative. For example, studies have compared the proportion of contaminated linen or karaya ostomy bags at one hospital to proportions at other hospitals [40], spore concentrations near a water leak to concentrations farther away [90], spores near construction to an area without construction [89], and spores at an air intake near the ground to other intakes [88]. While these studies have yielded some insight on environmental contamination, environmental sampling results should be interpreted with caution. They are best viewed as a supplement to a comprehensive environmental assessment and record review, rather than a replacement. More information is needed about what fungal species, if any, are seen in healthcare environments in the absence of an outbreak. 

### 2.9. Implement Control and Prevention Measures

Although it may not be possible to pinpoint the source of mold exposure during an outbreak, improving infection control practices can prevent future healthcare-associated infections. We recommend remediating all infection prevention gaps identified in the investigation. Support from hospital administration is key to the success of these interventions. Infection prevention should ensure that a risk assessment is performed prior to any construction or maintenance activities that may disperse dust. It is also important for infection prevention to collaborate with plant operations managers for the routine inspection of the condition of barriers used during construction, as well as airflow and air filtration from the construction site. Routine rounding in patient care areas should include proactive observation for water leaks that may become reservoirs for mold. Healthcare personnel should have competency-based training in the use of both negative and positive pressure rooms, including indications for the use of these rooms. A partnership between infection prevention and environmental services managers may be needed to identify new processes for handling clean linen to avoid environmental contamination prior to patient use.

Some facilities have implemented control measures that go beyond current recommendations in response to an outbreak. Some have given prophylactic antifungals effective against mucormycetes after considering the potential risks, patient tolerance, adverse effects, and cost. These extra measures have included expanding the use of respirators to patients who are immunosuppressed but did not receive HSCT, removing plants from areas outside protective environments, and using sterilized linen for some high-risk patients. To date, evidence is lacking to support or refute the efficacy of these expanded interventions for preventing mold infections in patients who have not had HSCT. Facilities considering interventions beyond the current recommendations can help expand the evidence by evaluating and publishing the results. For all interventions, prospective surveillance is needed to assess whether infections continue to occur. 

### 2.10. Initiate and Maintain Surveillance 

Ongoing, active surveillance of culture and histopathology specimens yields important information about whether interventions have been effective. Because they offer more detailed information, microbiology and pathology databases are recommended over diagnostic codes, such as International Classification of Diseases (ICD) codes. Although ICD codes may have low sensitivity, they can be used as a supplementary tool. When possible, infection control committees or outbreak review teams can include mucormycosis infections in their review. Medical record alerts may help for rapid notification. Ongoing collaborative surveillance across many hospitals in a region can also be invaluable in detecting when rates of infection are above baseline. 

## 3. Discussion

While not all investigations of mucormycosis infections will result in discovery of a single source, all can point to important areas for improvement in hospital infection control. Applying the tools in this guide will allow hospitals and public health partners to build on experience gained by other teams that have faced these challenging outbreaks. A comprehensive review of past infections can yield evidence on how potential sources of mold exposure may have changed over time, while ongoing surveillance provides valuable evidence on which interventions are effective. Rapid communication with treatment teams and patients can save lives through early identification of mucormycosis infections. Environmental assessments with partners in maintenance, construction, and environmental services can point to potential gaps in infection prevention and identify areas for remediation. All of these actions can improve patient safety, even if the source of mold is never definitively established. Continued efforts to understand the sources of mold that cause mucormycosis infections in healthcare will help prevent future infections and protect vulnerable patients. 

## Figures and Tables

**Table 1:** Case definition for invasive mucormycosis, adapted from the European Organization for Research and Treatment of Cancer (EORTC) and National Institute of Allergy and Infectious Diseases Mycoses Study Group (MSG); De Pauw et al. (2008) [21].

**Proven Invasive Mucormycosis:** Presence confirmed by pathology and/or culture of sterile material.	
Microscopic evidence	Histopathologic, cytopathologic, or direct microscopic examination of a specimen obtained by needle aspiration or biopsy in which characteristic hyphae are seen accompanied by evidence of associated tissue damage/vascular invasion. Mucormycete-specific microscopic findings may include: Broad, pauciseptate hyphae Large angle of branching, approaching right angles Irregular branching Angioinvasion Intravascular thrombosis
Culture evidence	Recovery of a mold by culture of a specimen obtained by a sterile procedure from a normally sterile and clinically or radiologically abnormal site consistent with an infectious disease process. Pathogenic mucormycete genera include but are not limited to: *Mucor, Rhizopus, Rhizomucor, Cunninghamella*, *Apophysomyces*, *Lichtheimia, Cokeromyces, Saksenaea*.
**Probable Invasive Mucormycosis:** Probable disease requires a host factor, a clinical criterion, and a mycological criterion, in the absence of confirmed non-mucormycete mold (e.g., *Aspergillus*).	
**Host factors**	Recent history of neutropenia (<500 neutrophils per microliter for >10 days) temporally related to the onset of fungal disease Receipt of allogeneic stem cell transplant Prolonged use of corticosteroids (mean minimum dose of 0.3 mg/kg/day of prednisone equivalent for >3 weeks) Treatment with other recognized T cell immunosuppressants, such as cyclosporine, TNF-alpha blockers, specific monoclonal antibodies, or nucleoside analogues during the past 90 days Inherited severe immunodeficiency (such as chronic granulomatous disease or severe combined immunodeficiency)
**Clinical criteria**	
Lower respiratory tract disease	Presence of one of the following three signs on CT: Dense, well-circumscribed lesion(s) with or without a halo sign Air-crescent sign Cavity
Tracheobronchitis	Tracheobronchial ulceration, nodule, pseudomembrane, plaque, or eschar seen on bronchoscopic analysis
Sinonasal infection	Imaging showing sinusitis plus at least one of the following three signs: Acute localized pain (including radiating to eye) Nasal ulcer with black eschar Extension from the paranasal sinus across bony barriers, including into the orbit
CNS infection	One of the following two signs: Focal lesion on imaging Meningeal enhancement on MRI or CT
**Mycological criteria**	Mold in sputum, bronchoalveolar lavage fluid, bronchial brush, or sinus aspirate samples indicated by one of the following: Presence of fungal elements indicating a mold Recovery by culture of a mold
**Possible Invasive Mucormycosis:** Possible disease requires a host factor and a clinical criterion from **Probable Invasive Mucormycosis** above.	

**Table 2:** Attributed or suspected healthcare exposures in published reports of mucormycosis.

Exposure Reported as Likely Cause of Infection	Number of Infections and Site(s)	Publication Year	Reference
**Non-Sterile Products**			
**Bandages, patches, tape, and adhesives**			
Elastic adhesive bandages	>20 cutaneous	1978–1980	[44,45,46,47,48,49,50]
Bandages	5 wounds; 3 necrotizing fasciitis	2005, 2011	[51,52]
Tape used to secure endotracheal tube	2 cutaneous	1998, 2002	[53,54]
Karaya gum adhesive used to secure ostomy bag	2 cutaneous infections of stomas	2006	[40]
Adhesive used to secure heart monitor and temperature probe in preterm infants	2 cutaneous	1997	[55,56]
Nitroglycerin patches applied at home	1 cutaneous	2003	[57]
Fabric under a cast	1 cutaneous	1993	[58]
**Linen**			
Contamination of linen and linen delivery bins	5 cutaneous	2014	[17]
Contamination of hospital laundry carts	4 cutaneous	2016	[59]
Poor ventilation, high humidity, and dust at company supplying hospital linen	3 pulmonary, 2 cutaneous, 1 pulmonary and cutaneous	2016	[18]
**Wooden tongue depressors**			
Wooden tongue depressors used to prepare oral medications given through nasogastric catheter	5 gastric	2004	[60]
Wooden tongue depressors used as splints	4 cutaneous	1996	[61]
**Medications, supplements, and food**			
Probiotic powdered supplement	1 gastrointestinal	2014	[43]
Probiotic oral supplement	1 hepatic	1996	[62]
Allopurinol tablets	17 intestinal	2009	[63]
**Procedures, Devices, and Organs**			
**Catheters and tubes**			
Permanent bladder catheter	1 bladder	2004	[64]
Periumbilical cutaneous catheter and chest tube	1 cutaneous; 1 chest wall and pulmonary	1998	[65]
Orogastric tube	1 gastrointestinal	2004	[66]
Catheter for continuous ambulatory peritoneal dialysis	4 peritonitis	1989, 1991, 2003, 2006	[67,68,69,70]
**Dental procedures**			
Unspecified dental procedure	2 rhino-orbital; 1 rhinocerebral	2006, 2009	[71,72]
Tooth extraction	1 mandible, 1 rhinocerebral, 1 maxilla, 1 jaw	1997, 2001, 2006, 2018	[73,74,75,76]
**Cardiac surgery**			
Explantation of left ventricular assist device	1 aortic	2008	[77]
Implantation of prosthetic valves	3 prosthetic valves	1972, 1987, 1999	[78,79,80]
Catheterization and bypass surgery	1 sternotomy wound	1994	[81]
**Insulin pumps and finger sticks**			
Insulin infusion pump insertion	1 cutaneous	1989	[82]
Daily insulin needle punctures	2 cutaneous	2004, 2017	[83,84]
Finger sticks for blood glucose self-monitoring	1 disseminated (initially cutaneous)	2005	[85]
**Medical devices**			
Deficiencies in sterilization of screws used in ligament reconstruction	3 bone	2016	[86]
**Organ transplant**			
Kidneys donated by near-drowning victim after motor-vehicle crash	2 renal	2010	[41]
**Environmental Sources: Air, Dust, Water, Soil**			
**Air filtration and intakes**			
Air filter insufficient to trap spores	4 mycotic endocarditis, including one with *Mucor* sp.	1992	[87]
Air intake close to the ground in outdoor yard	3 sinus	1993	[88]
**Negative pressure room**			
Use of negative-pressure isolation room for transplant surgery patients	1 disseminated, 1 cutaneous, 1 pulmonary	2015	[42]
**Construction**			
Inadequate barriers during renovation	2 pulmonary	1985	[89]
**Leaks and water damage**			
Shower leak that led to mold growth on wall in linen closet near patient rooms	1 brain and 1 cutaneous	2008	[90]
**Plants**			
Petal, stem, and leaf applied to skin for allergy test	1 cutaneous	2002	[91]

## Supplementary Materials

The following are available online at https://www.mdpi.com/2309-608X/5/3/69/s1, S1: Common pathogenic mold species. S2: Sample line list of variables to collect for mucormycosis. S3: Targeted environmental investigation checklist for outbreaks of invasive infections caused by environmental fungi.
