# Supplementary material for: A Guide to Investigating Suspected Outbreaks of Mucormycosis in Healthcare
Source: J Fungi (Basel). 2019 Jul 24;5(3):69. doi: 10.3390/jof5030069 (PMC6787571; doi:10.3390/jof5030069)
Supplement: Supplementary file 1 [file jof-05-00069-s001.zip › Supplementary_Materials_S3.docx]

**Targeted Environmental Investigation Checklist for Outbreaks of Invasive Infections Caused by Environmental Fungi (*e.g.*, *Aspergillus*, mucormycetes)**

This tool is intended to aid in performing an environmental assessment when investigating healthcare-associated disease outbreaks caused by certain fungi. This checklist is most relevant for environmental fungi like *Aspergillus* spp. and mucormycetes. It is less relevant for fungi like *Candida auris* that involve skin colonization and disperse through inadequate hand hygiene and environmental disinfection; assessment of adherence to infection control practices and environmental cleaning and disinfection are typically more relevant for those investigations.

Epidemiologic assessment should be the foundation of any investigation into outbreaks caused by environmental fungi. Environmental sampling may also be conducted, but sampling should be guided by epidemiologic findings and the environmental assessment, with an understanding that the implications of this testing are unlikely to be definitive.

Not all of sections of this checklist will pertain to every investigation. Where possible, use epidemiologic evidence to guide which sections to complete. Please use the notes section to describe key findings and concerns. When possible, use diagrams and photos to document the locations and findings of the assessment.

**Table of Contents**

[Section 1: Initial observations and visual inspection 2](#_Toc5017196)

[Walk-through inspection 2](#_Toc5017197)

[Recent events involving the built environment (up to six months prior to first case) 2](#_Toc5017198)

[Section 2. Heating, ventilation and air conditioning (HVAC) system 3](#_Toc5017199)

[Heating, ventilation and air conditioning (HVAC) system 3](#_Toc5017200)

[Air changes 3](#_Toc5017201)

[Air intakes (from exterior) 3](#_Toc5017202)

[Filters 4](#_Toc5017203)

[HVAC system design & components 4](#_Toc5017204)

[Ductwork diffusers and grille plates 5](#_Toc5017205)

[Pressurization and air flow 5](#_Toc5017206)

[Section 3: Built environment and environmental services 5](#_Toc5017207)

[General 5](#_Toc5017208)

[Environmental cleaning processes 5](#_Toc5017209)

[Service area(s) 6](#_Toc5017210)

[Indoor public areas (e.g. atria, corridors) 6](#_Toc5017211)

[Section 4: Laundry and healthcare textiles (HCT) 7](#_Toc5017212)

[HCT storage rooms 7](#_Toc5017213)

[HCT bundles 7](#_Toc5017214)

[Laundry operations 8](#_Toc5017215)

[Section 5: Equipment and medical devices (if implicated as a possible source of exposure) 9](#_Toc5017216)

[Section 6: Construction, renovation, demolition, and repair 9](#_Toc5017217)

[Internal construction 9](#_Toc5017218)

[At-risk patients 10](#_Toc5017219)

[External construction 10](#_Toc5017220)

[Section 7: Protective environments (PE) and at-risk patients 11](#_Toc5017221)

[Abbreviations: 11](#_Toc5017222)

[Source documents and key references: 11](#_Toc5017223)

# Section 1: Initial observations and visual inspection

| Walk-through inspection **(select patient care areas and other locations based on relevance to potential patient exposures, as informed by epidemiologic review of initial cases)**  ***Include the following areas in observations: windows, doors, ceilings, walls, floors, bathroom water fixtures/drains, beds including mattress and linens and furnishings*** | |
| --- | --- |
| **Elements to be assessed** | **Notes (include date, location affected, and facility elements affected)** |
| - General cleanliness (visible dust, dirt, stains, etc.) |  |
| - Water damage present (stains, dampness, leaks around plumbing, leaks in ceiling, condensation on surfaces) |  |
| - Evidence of fungal growth and damage (visible mold and mildew, stains, mold odor, fungal growth on drywall surface or underneath wall coverings, fungal growth on building’s interior support structures and building materials) |  |
| - Evidence of air intrusion from outdoors or from other adjacent spaces (note the status of window seals, seals surrounding plumbing pipe intrusions, lack of self-closing doors) |  |
| - Other observations of disrepair, lack of maintenance |  |
| Recent events involving the built environment (up to six months prior to first case) | |
| **Elements to be assessed** | **Notes** |
| - System disruptions (e.g., maintenance, water shut-offs, power outages) |  |
| - Internal construction, renovation, demolition, repairs |  |
| - External construction, renovation, demolition, repairs |  |
| - Flooding, heavy rains, or other water damage to facility   - If yes, did the facility take steps to dry out wet structural materials within 2-3 days?   - If yes, did the facility remove any wet structural materials that could not be dried out within the 2-3 day period? |  |

# Section 2. Heating, ventilation and air conditioning (HVAC) system

| Heating, ventilation and air conditioning (HVAC) system | |
| --- | --- |
| **Elements to be assessed** | **Notes** |
| - HVAC system characteristics including number of systems serving the area, ambient climate control, airflow direction, pressure readings, number of air changes per hour (ACH), and status of filters |  |
| - HVAC maintenance and repair including schedule and practices for: change out of filters, filter frame inspection, air duct cleaning, air handler coil maintenance, drip pan maintenance, visual assessment of supply air grilles for dust accumulation |  |
| - Schedule and strategy for building and HVAC system routine maintenance and repair |  |
| - Building construction date (or when was the last major renovation involving the HVAC system) |  |
| - Notable events or air flow disturbances related to the HVAC system |  |
| Air changes |  |
| For each area/location investigated:   - Total mechanical supply air changes per hour (specify in notes) - Total mechanical exhaust air changes per hour (specify in notes) - Outdoor air changes per hour (specify in notes, indicate how determined)   NOTE: If Variable Air Volume and/or setback operations are used, record above parameters for both “normal” and reduced flow rates and document their operational sequence. |  |
| Air intakes (from exterior) |  |
| For each area/location investigated, assess: |  |
| - Intakes located at least 25 feet from cooling towers and away from potential contaminants (auto traffic, outdoor construction) |  |
| - Intakes at least 25 feet from exhaust outlets   * Exception: For gas-fired, packaged rooftop units, distance from outdoor air intake from flue may be <25 ft; see ANSI/ASHRAE Standard 62.1, Table 5-1 for distances applicable for these units. |  |
| - Intakes at least 6 ft above grade or 3 ft above roof |  |
| - Evidence of animals, fungal growth, or standing water around air intakes |  |
| - Intakes have features to drain away precipitation |  |
| - Intakes equipped with mesh birdscreen |  |
| - If construction site nearby or if air intake is downwind of construction, does facility have a plan to address air during construction |  |
| - Plan in place to shut down HVAC or monitor rough-in or pre-filter, if the air intake is near construction |  |
| - Air mixing box functioning correctly |  |
| Filters |  |
| For each area/location investigated, assess:   - Filter efficiency (MERV and/or HEPA) - Location of the filter (room or area) |  |
| - Filtration efficiency and performance complies with FGI standards |  |
| - Frequency of filter performance monitoring (specify in notes) - Method used to monitor filter performance (specify in notes) |  |
| - Filter situated in frame so that air does not bypass filter |  |
| - First filtration bank is upstream of heating and cooling coils |  |
| - Second filtration bank is downstream from wet-air cooling coils and supply fan |  |
| - Filters (including rough-in and pre-filters) in good repair without excess dust and debris |  |
| - Final filter for each fan unit intact |  |
| - Filters and fans maintained according to manufacturer’s instructions (review maintenance log) |  |
| - Seal in place for fan and filter access doors |  |
| HVAC system design & components |  |
| - Fans in good condition and operational |  |
| - Type of cooling system (specify in notes) |  |
| - Presence/type/set points for humidification system (specify in notes) |  |
| - Review cooling coils maintenance plan and observations from last inspection |  |
| - Cooling coil drip pans clean (e.g., no evidence of standing water) |  |
| - Room average temp (winter/summer; specify) |  |
| - Room average relative humidity (winter/summer; specify) |  |
| - Are there documented episodes where humidity exceeds acceptable upper limit? (see FGI quick reference) If yes, describe frequency |  |
| Ductwork diffusers and grille plates |  |
| - Ductwork, diffusers, and grille plates unobstructed (i.e. not blocked by furniture or equipment) |  |
| - If space reconfigured, are diffuser locations appropriate for the current workspace? |  |
| - Ductwork, diffusers, and grille plates clean and free of excess dust and debris |  |
| - Insulation dry and free of visible mold/mildew |  |
| - Insulation more than 15 feet downstream of humidifiers |  |
| - Air supply diffusers not redirected? |  |
| - Exhaust in good condition and functioning properly? |  |
| - Shadow present on the ceiling along the air pathway away from the supply grille |  |
| - Supply air grille located in the room |  |
| - Room a laminar flow room (if yes, indicate horizontal or vertical) |  |
| Pressurization and air flow |  |
| - Airflow direction & magnitude (provide map and specify direction & magnitude in notes) |  |
| - Continuous visual and/or engineered (e.g. manometer) monitoring of pressure and air flow direction |  |

# Section 3: Built environment and environmental services

| General | |
| --- | --- |
| **Elements to be assessed** | **Notes** |
| - Year building was constructed (specify in notes) |  |
| - Dates of renovations of outbreak space (please list dates in notes) |  |
| - Evidence or history of interstitial water intrusion |  |
| - Roof composed of hard, non-organic materials (i.e. not a green roof) |  |
| - Roof undergoing repairs or construction |  |
| - Roof free of leaks   If no to above, note location of leaks and estimated length of time it was present   - - Have the leak(s) been repaired? |  |
| Environmental cleaning processes |  |
| - Facility has established and follows a schedule for areas and equipment to be cleaned and serviced regularly |  |
| - Cleaning solutions prepared daily or as needed |  |
| - Separate clean cloths used for each room |  |
| - Mop heads and cleaning cloths laundered daily |  |
| - Rooms cleaned and disinfected daily and terminally as needed   If yes, indicate EPA disinfectant used  If no, specify cleaning schedule |  |
| - Surfaces wet dusted |  |
| - Cleaners and disinfectants used according to manufacturer’s instructions |  |
| - Furniture with smooth surfaces cleaned |  |
| - Privacy curtain replaced or cleaned with scheduled frequency (specify schedule, if possible) |  |
| - In protected environments (positive pressure rooms), EVS staff wear gowns and gloves while cleaning in these rooms |  |
| Service area(s) *Include the following areas in observations: mechanical rooms, storage areas corridors, janitorial areas, elevators, and stairwells* | |
| - Free of water damage, dampness, dust/debris, visible mold/mildew |  |
| - Boilers or steam-producing equipment located away from patient care areas |  |
| - Areas adjacent to rooms with boilers or steam-producing equipment dry (i.e. no visible condensation) |  |
| - Critical care equipment storage and patient care areas separated |  |
| - Storage for patient care items located away from loading docks |  |
| Indoor public areas (e.g. atria,corridors) | |
| - Free of water damage, dampness, dust/debris, visible mold/mildew |  |
| - All surfaces able to be reached for cleaning   - If no, plan in place to address hard- to-reach surfaces |  |
| - Area free of water features (e.g., fountains, water walls, etc.) and fish tanks   - If no, polices in place to ensure regular cleaning and/or disinfecting of water features |  |
| - If the corridor floor is carpeted, it is regularly vacuumed and/or shampooed (describe frequency) |  |
| - Non-care rooms (e.g., conference rooms) immediately adjacent to patient care-areas   - If yes, are these rooms carpeted   - If yes, describe vacuum/shampoo frequency in notes |  |

#

# Section 4: Laundry and healthcare textiles (HCT)

| HCT storage rooms |  |
| --- | --- |
| **Elements to be assessed** | **Notes** |
| Specify location of inspection:   - Hospital - Contract laundry - Other(please explain in notes) |  |
| - HCT storage room located away from facility loading dock |  |
| - Clean HCT storage room under positive pressure |  |
| - Protocols to minimize dust entry at loading dock |  |
| - Storage room has self-closing doors that are kept closed |  |
| - Room traffic kept to a minimum |  |
| - Surfaces clean and free of dust |  |
| - Rooms cleaned and disinfected regularly |  |
| Room temp (winter/summer; specify) |  |
| Relative humidity (winter/summer; specify) |  |
| ACH per hour (specify) |  |
| Airflow direction (specify) |  |
| HCT bundles |  |
| - HCT bundles covered when stored |  |
| - Wrapped HCT bundles are dry (i.e., not moist to the touch) |  |
| - If HCT bundles not wrapped, they are protected from dust |  |
| - Carts used to transport HCT are clean |  |
| - Carts protected from contamination while in storage or during transit |  |
| - Bed/bath HCT changed on regular schedule (indicate frequency; e.g, daily, weekly) |  |
| - EVS staff perform hand hygiene before changing HCT in patient rooms |  |
| - HCTs changed when wet in a timely fashion (i.e. do not stay wet for several hours) |  |
| - Personnel handle soiled HCT with personal protective equipment (PPE) and with minimum agitation |  |
| - Contaminated HCT bagged or contained at point of use |  |
| - Contaminated HCT labeled, color-coded, or otherwise marked |  |
| - Linens indicated for use on specialty beds laundered by the hospital’s laundry provider |  |
| - Hospital uses a laundry chute to move soiled textiles |  |
| Laundry operations |  |
| - Laundry services provided by contractors other than the healthcare facility |  |
| - Laundry equipment used and maintained according to manufacturer’s instructions |  |
| - Dryers have in-line filters on the incoming air source |  |
| - Sanitizers and disinfectants used in wash cycles |  |
| - Processes in place to ensure HCT do not touch floor during laundering |  |
| - Water recycling procedures are used |  |
| - Wash and dry process procedures are specified (i.e., wash water temperatures, dryer temperatures) |  |
| - HCT are dry to the touch when bundled |  |
| - Typical holding time for bundled HCT prior to transport to facility is short (specify average hold time if possible) |  |
| - Facility is clean (i.e., no visible dust or lint) |  |
| - No lint release visible on roof |  |
| - Receiving and soiled sort area are at negative pressure |  |
| - Areas where clean textiles are manipulated are at positive pressure |  |
| - Laundry areas have handwashing facilities /products and appropriate PPE for workers |  |
| - Clean laundry protected from dust and lint during blowdown |  |
| - Outdoor air infiltration allowed inside the plant (if yes, determine why this is allowed |  |
| - Does the laundry have a packroom for surgical textiles |  |
| - Storage/holding area for sterilized HCT separate from clean holding area |  |
| - Bundled HCT held indoors prior to transport |  |
| - Truck cargo bay clean and door closes properly |  |
| - Truck cargo bays provide for separation of clean and dirty loads |  |

# Section 5: Equipment and medical devices

| **Elements to be assessed** | **Notes** |
| --- | --- |
| For each relevant piece of equipment or device implicated as a possible source of exposure:  Check for presence of water, condensate, dust, fans and filter status (if applicable). |  |
| Check cleaning and disinfection strategy and practice. List devices assessed and findings in notes. |  |

# Section 6: Construction, renovation, demolition, and repair

| **Elements to be assessed** | **Notes** |
| --- | --- |
| Internal construction |  |
| - Internal construction, renovation, demolition or repairs in previous 6-12 months prior to outbreak (or as appropriate for fungal agent)   If yes, record date, location, and facility elements affected |  |
| - External construction, renovation, demolition or repairs in previous 6-12 months prior to outbreak (or as appropriate for fungal agent)   If yes, record date location and facility elements affected. |  |
| - Facility conducted an ICRA prior to start of construction   - If yes, ICRA documentation available for review |  |
| - Negative air pressure in construction zone maintained and verified |  |
| - Adjacent spaces under positive pressure |  |
| - Portable HEPA filters used if needed for dust control in construction zone   - If yes, HEPA filter units tested & functioning |  |
| - Exhaust from construction zone directed away from air intakes |  |
| - Plans developed to manage removal of debris, dust control at entrance/exit, and construction personnel clean up |  |
| - Carts with construction debris covered |  |
| - Demolition managed to minimize dust dispersion |  |
| - Impermeable barriers effectively maintained negative pressure in construction zone |  |
| - Plastic sheets or other barriers used   - If yes, note location and type |  |
| - Action plan in place if there are infection control breaches identified |  |
| - Breaches in infection control associated with the construction project been identified   - If yes, describe what was done to correct them |  |
| - Construction personnel diverted away from patient care areas   - If no, is there space for changing clothing, equipment, access to food service |  |
| - If equipment or tools leave construction zone, are they vacuumed or wet-dusted to control dust |  |
| - Building materials in good condition with no evidence of physical and/or water damage, dampness, dust, visible mold, or mildew |  |
| At-risk patients |  |
| - Construction does not directly involve at-risk patient area |  |
| - Protected environment patients relocated away from construction and construction traffic areas |  |
| - Construction occurring in areas separate from (i.e., not adjacent to) at risk patient care areas |  |
| - HVAC system serving at-risk patient care areas are independent or isolated from remainder of HVAC system |  |
| External construction |  |
| - Is the hospital directly adjacent to construction project   - If yes, air intakes protected or sealed |  |
| - Strategy in place to prevent overloading of HVAC filters |  |
| - Windows are well sealed |  |
| - Pedestrian entrances facing construction are closed |  |
| - Loading dock redirected if facing construction areas |  |
| - Water is monitored for contamination from construction |  |

# Section 7: Protective environments (PE) and at-risk patients

| **Elements to be assessed** | **Notes** |
| --- | --- |
| Filter bank efficiency:   - First filter bank: MERV 7 minimum - Second filter bank with minimum HEPA efficiency is present OR second filter bank with MERV-14 filters if a tertiary terminal filter with minimum HEPA efficiency is provided for these spaces |  |
| - Filters situated in frames or housing |  |
| - 12 total (minimum) air changes per hour (ACH) |  |
| - Relative humidity ≤ 60% |  |
| - Positive pressure maintained per FGI and monitored/recorded daily |  |
| - Juncture where ceiling meets walls and walls meet floor are sealed and continuous? |  |
| - Supply air diffuser located at or near ceiling |  |
| - Planned redundancy for ventilation supplying PE |  |
| - No carpeting or upholstered furniture used in patient room or care areas |  |
| - No fresh or dried flowers or potted plants in patient care area |  |
| - Allogenic patients wear N95 outside of PE, if needed (e.g. construction in other areas) |  |
| - Water leaks cleaned and repaired within 72 hours |  |

# Abbreviations:

ACH: Air changes per hour

ASHRAE: American Society of Heating, Refrigerating, and Air-Conditioning Engineers

EPA: US Environmental Protection Agency

EVS: Environmental services

FGI: Facility Guidelines Institute

HCT: Healthcare textiles

HEPA: High efficiency particulate air

HVAC: Heating, ventilation, air conditioning

ICRA: Infection control risk assessment

MERV: Minimum efficiency reporting value

# Source documents and key references:

FGI Guidelines, 2014 <https://www.fgiguidelines.org/guidelines/2014-fgi-guidelines/>

FGI Guidelines, 2018 <https://www.fgiguidelines.org/guidelines/2018-fgi-guidelines/>

ASHRAE standard 170, 2013 <https://www.techstreet.com/ashrae/standards/ashrae-170-2013?product_id=1869692>

ASHRAE standard 170, 2017 <https://www.techstreet.com/ashrae/standards/ashrae-170-2017?product_id=1999079&ashrae_auth_token=12ce7b1d-2e2e-472b-b689-8065208f2e36>

CDC Environmental Infection Control Guidelines, 2003 <https://www.cdc.gov/infectioncontrol/guidelines/environmental/index.html>

CMS Hospital Infection Control Worksheet, 2014 <https://www.cms.gov/medicare/provider-enrollment-and-certification/surveycertificationgeninfo/downloads/survey-and-cert-letter-15-12-attachment-1.pdf>

ICRA template: American Society of Healthcare Engineers, see <http://www.ashe.org/resources/tools/pdfs/assessment_icra.pdf>

ILSM template: American Society of Healthcare Engineers, see <http://www.ashe.org/resources/tools/pdfs/ilsm-icra_monitor.pdf>

Kanamori et al., 2015. *Clinical Infectious Diseases.* Aug 1; 61(3). <https://www.ncbi.nlm.nih.gov/pubmed/25870328>

Tomblyn et al., 2009. *Biol Blood Marrow Transplant.* Oct; 15(10). <https://www.ncbi.nlm.nih.gov/pmc/articles/PMC3103296/>

Guidelines for preventing opportunistic infections among hematopoietic stem cell transplant recipients, 2000 <https://www.cdc.gov/mmwr/preview/mmwrhtml/rr4910a1.htm>
